# CHAC1 overexpression in human gastric parietal cells with *Helicobacter pylori* infection in the secretory canaliculi

**DOI:** 10.1111/hel.12598

**Published:** 2019-05-20

**Authors:** Tomohisa Ogawa, Yuriko Wada, Kosuke Takemura, Philip G. Board, Keisuke Uchida, Keisuke Kitagaki, Tomoki Tamura, Takashige Suzuki, Yutaka Tokairin, Yasuaki Nakajima, Yoshinobu Eishi

**Affiliations:** ^1^ Department of Human Pathology, Graduate School and Faculty of Medicine Tokyo Medical and Dental University Tokyo Japan; ^2^ Department of Urology Tokyo Metropolitan Cancer and Infectious Diseases Center Komagome Hospital Tokyo Japan; ^3^ The ACRF Department of Cancer Biology and Therapeutics, Group of Molecular Genetics The John Curtin School of Medical Research, Australian National University Canberra Australian Capital Territory Australia; ^4^ Division of Surgical Pathology Tokyo Medical and Dental University Hospital Tokyo Japan; ^5^ Department of Gastrointestinal Surgery Tokyo Medical and Dental University Tokyo Japan

**Keywords:** cation transport regulator 1, gastric cancer, *Helicobacter pylori*, parietal cells, secretary canaliculi

## Abstract

**Background:**

Cation transport regulator 1 (CHAC1), a newly discovered enzyme that degrades glutathione, is induced in *Helicobacter pylori* (*H. pylori*)‐infected gastric epithelial cells in culture. The CHAC1‐induced decrease in glutathione leads to an accumulation of reactive oxygen species and somatic mutations in *TP53*. We evaluated the possible correlation between *H. pylori* infection and CHAC1 expression in human gastric mucosa.

**Materials and Methods:**

Both fresh‐frozen and formalin‐fixed paraffin‐embedded tissue samples of gastric mucosa with or without *H. pylori* infection were obtained from 41 esophageal cancer patients that underwent esophago‐gastrectomy. Fresh samples were used for real‐time polymerase chain reaction for *H. pylori* DNA and CHAC1 mRNA, and formalin‐fixed samples were used for immunohistochemistry with anti‐CHAC1 and anti‐*H. pylori* monoclonal antibodies. Double‐enzyme or fluorescence immunohistochemistry and immuno‐electron microscopy were used for further analysis.

**Results:**

Significant CHAC1 overexpression was detected in *H. pylori*‐infected parietal cells that expressed the human proton pump/H,K‐ATPase α subunit, whereas a constitutively low level of CHAC1 mRNA expression was observed in the other samples regardless of the *H. pylori* infection status, reflecting the weak CHAC1 expression detected by immunohistochemistry in the fundic‐gland areas. Immuno‐electron microscopy revealed intact *H. pylori* cells in the secretory canaliculi of infected parietal cells. Some parietal cells exhibited positive nuclear signals for Ki67 in the neck zone of the gastric fundic‐gland mucosa with *H. pylori* infection.

**Conclusion:**

Cation transport regulator 1 overexpression in *H. pylori*‐infected parietal cells may cause the *H. pylori*‐induced somatic mutations that contribute to the development of gastric cancer.

## 1 INTRODUCTION

Cation transport regulator 1 (CHAC1) is a newly discovered enzyme involved in the γ‐glutamyl cycle that can degrade glutathione (GSH) into 5‐oxoproline and cysteinyl‐glycine.^1^, ^2^ CHAC1 is a constituent of the unfolded protein response stress signaling pathway in the endoplasmic reticulum (ER),^3^, ^4^ and as a consequence of ER stress, the increase in CHAC1 leads to depletion of GSH and results in unbalanced cellular redox levels.^5^

Endoplasmic reticulum stress is triggered by various stimuli, such as infection, inflammation, and gene mutations,^6^ and is widely related to the development of several malignant tumors.^7^ *Helicobacter pylori* (*H. pylori*) infection can mediate ER stress,^8^ drastically decreasing GSH levels and increasing the production of reactive oxygen species (ROS) in gastric epithelial cells.^9^, ^10^, ^11^ We recently reported that CHAC1 expression is an essential factor driving these sequential changes in infected cells.^12^ The decreased GSH and increased ROS lead to somatic mutations in *TP53*, suggesting that *H. pylori*‐induced CHAC1 overexpression in infected gastric epithelial cells directly contributes to gastric cancer.^12^

*Helicobacter pylori* is a gram‐negative, micro‐aerophilic spiral‐shaped bacterium that colonizes the gastric mucosa of the human stomach. More than half of the human population worldwide is infected with *H. pylori*.^13^ Immunohistochemistry (IHC) with a novel anti‐*H. pylori* monoclonal antibody (TMDU‐mAb) revealed that *H. pylori* can be detected not only in the mucus layer attached to the superficial gastric foveolar cells, but also in macrophages scattered in the lamina propria and notably in some parietal cells in *H. pylori*‐infected gastric mucosa.^14^ We recently reported that *H. pylori* infection promotes CHAC1 expression,^12^ but the specific gastric cell types where this occurs in vivo in response to *H. pylori* infection were not clarified. In the present study, we examined gastric mucosa with or without *H. pylori* infection by real‐time reverse transcription polymerase chain reaction (PCR) for CHAC1 mRNA using fresh‐frozen tissues and by IHC with a novel anti‐CHAC1 monoclonal antibody (CHAC1‐mAb_(v1v2)_) to locate cells with CHAC1 overexpression in formalin‐fixed paraffin‐embedded (FFPE) tissue sections.

## 2 MATERIALS AND METHODS

### 2.1 Human tissue samples

To investigate CHAC1 expression in the gastric mucosa with or without *H. pylori* infection, we collected samples from patients with esophageal cancer that underwent esophago‐gastrectomy of the proximal one‐third of the stomach and lower half of the esophagus. This study was designed according to our previous result that *H. pylori* infection was detected by PCR or IHC in many samples from the corpus of the stomach with gastric cancer.^14^ Both fresh‐frozen and 10% neutral buffered FFPE tissue samples of the gastric mucosa were obtained from 17 patients with *H. pylori* infection and 24 patients without *H. pylori* infection between January 2013 and December 2016 at Tokyo Medical and Dental University Hospital. The fresh fundic‐gland mucosa was mounted in Tissue‐Tek OTC Compound (Sakura Finetek Japan), quick‐frozen in liquid nitrogen, and maintained −70°C until use.

The clinical profiles of the patients and *H. pylori* infection status of each sample are shown in Table S1. The *H. pylori* infection status of each sample was estimated by enzyme IHC for the bacterium using FFPE tissue sections and real‐time PCR for the bacterial 16S ribosomal RNA^14^ using frozen sections. All patients received an explanation regarding the purpose of the study and provided written informed consent to participate in the study. The Tokyo Medical and Dental University ethics committee approved this study (Registration No. 1706). All methods were performed in accordance with the relevant guidelines and regulations.

### 2.2 DNA extraction and real‐time PCR

For DNA extraction, 60‐µm‐thick fresh‐frozen tissue sections were treated with TaKaRa DEXPAT^™^ Easy (Takara Bio Inc,) according to the manufacturer's instructions. Fragments of 16S ribosomal RNA of *H. pylori* were amplified by real‐time PCR using TaqMan Universal PCR Master Mix (ABgene). The primers and probes used for this assay are shown in Table S2. Amplification and detection were performed using the ABI PRISM 7900HT Sequence Detection System (Applied Biosystems). The amount of *H. pylori* DNA was expressed in terms of the number of bacterial genomes, with 1.25 × 10^10^ Da per genome used for the conversion. Negative controls without bacterial DNA were included in every PCR assay; background values were always <1 genome. Samples with one or more bacterial genome were considered positive. The total number of *H. pylori* in each sample was calculated by multiplying the assay results by 40.

### 2.3 RNA extraction and real‐time reverse transcription‐PCR

To extract RNA, 60‐µm‐thick fresh‐frozen tissue sections were treated with 1.0 mL of TRIzol^®^ reagent (Invitrogen) according to the manufacturer's instructions. cDNA was synthesized with random primers using Superscript^™^ Reverse Transcriptase (Invitrogen). The oligonucleotide primers and probes are listed in Table S2. The relative mRNA quantification was determined by real‐time reverse transcription‐PCR using the TaqMan Universal PCR Master Mix (ABgene). Amplification and detection were performed with the ABI PRISM 7900HT Sequence Detection System (Applied Biosystems). The amount of target cDNA was normalized with that of the endogenous mRNA of either the housekeeping reference β‐actin or the human proton pump/H,K‐ATPase α subunit for fresh‐frozen stomach tissues.

### 2.4 Enzyme Immunohistochemistry

Histologic sections (3‐µm‐thick) cut from FFPE tissue samples were mounted on silane‐coated slides (Muto Pure Chemicals Co. Ltd.), de‐paraffinized, rehydrated, and pretreated with the appropriate antigen retrieval methods for each antigen. The sections were microwaved for 40 minutes at 97°C to detect CHAC1, *H. pylori*, and proton pump, or autoclaved for 20 minutes at 121°C to detect the Ki67 antigen, both in 10 mM citrate buffer (pH 6.0). TMDU‐mAb and CHAC1‐mAb_(v1v2)_ are described elsewhere, respectively.^12^, ^14^ The proton pump/H,K‐ATPase α subunit, “proton pump,” is considered a specific marker of acid‐secreting parietal cells.^15^

Sections were treated with 3% hydrogen peroxide in methanol for 10 minutes, and then incubated with normal horse serum for 10 minutes (Vectastain Universal Elite ABC Kit; Vector Laboratories). The sections were then incubated with either of the appropriately diluted first antibodies (CHAC1‐mAb_(v1v2)_, TMDU‐mAb, anti‐proton pump antibody [D031, MBL], or Ki‐67 antibody [M7240, DAKO]) overnight at room temperature. Following incubation with biotinylated horse secondary antibody, the sections were incubated with streptavidin‐peroxidase complex for 30 minutes at room temperature (Vectastain Universal Elite ABC Kit). Before and after each step, the sections were washed with PBS containing 0.25% Tween‐20. Diaminobenzidine (Histofine Simplestain DAB Solution; Nichirei Bioscience) was used as the chromogen. All specimens were counterstained with Mayer's hematoxylin. Adjacent sections were stained with hematoxylin and eosin staining for further histologic examination.

### 2.5 Double‐enzyme and fluorescence Immunohistochemistry

Histologic sections were processed in the same manner as for enzyme IHC up to the primary antibody reaction. The sections were incubated with alkaline phosphatase‐conjugated secondary antibody followed by the VECTOR Blue Alkaline Phosphatase Substrate Kit III (SK‐5300, Vector Laboratories) or with biotinylated horse secondary antibody (Vectastain Universal Elite ABC Kit), and then incubated with fluorescein isothiocyanate‐conjugated streptavidin (F0250, DAKO). The sections were then microwaved in 10 mmol/L citrate buffer (pH 6.0) for 20 minutes to inactivate the antibodies used for detecting the first antigen, and then incubated with normal horse serum for 10 minutes. Subsequently, the sections were incubated overnight with the appropriately diluted first antibodies against the second antigen at room temperature. The sections were next incubated with EnVision + System‐HRP Labelled Polymer (K4001, DAKO) followed by the Histofine Simplestain DAB Solution, or with tetramethylrhodamine isothiocyanate‐conjugated anti‐mouse immunoglobulin (R0270, DAKO). Double fluorescence IHC was performed using a fluorescence laser‐scanning microscope (FV1200; Olympus).

### 2.6 Immuno‐electron microscopy

Histologic sections were processed in the same manner as for IHC until the signal development against *H. pylori* by DAB. The sections were washed several times with 0.1 mol/L phosphate buffer (pH 7.4), and fixed with 2.5% glutaraldehyde in 0.1 mol/L phosphate buffer for 60 minutes at 4°C, followed by post‐fixation with 1% osmium tetroxide for 60 minutes at room temperature. The sections were then dehydrated and embedded in Epon 812 (TAAB Laboratories Equipment Ltd.,). For flat embedded specimens, gelatin capsules filled with Epon were placed on top of section areas that were selected based on parallel sections treated for enzyme IHC. After polymerization, the Epon blocks containing the tissue were peeled from the glass slides by heating, and trimmed precisely with small tissue areas (1 mm^2^) readily identifiable on the block surface under reflective light. A Reichert Ultracut S (Leica EM UC7 Microsystems, Heidelberg GmbH) was used to cut ultrathin sections that were collected on Maxtaform grids (Pyser‐SGL Ltd.,). The sections were stained with uranium and lead citrate and examined under an H‐7700 electron microscope (Hitachi High‐Technologies Co.,).

### 2.7 Statistical analysis

Statistical analysis was performed using GraphPad PRISM ver. 6 (GraphPad Software, Inc,). Differences in the CHAC1 mRNA expression between samples with and without *H. pylori* infection were assessed using the Mann‐Whitney *U* test. A two‐tailed *P*‐value of <0.05 was considered statistically significant.

## 3 RESULTS

### 3.1 Cation transport regulator 1 expression in *Helicobacter pylori*‐infected gastric mucosa

**Figure 1:**
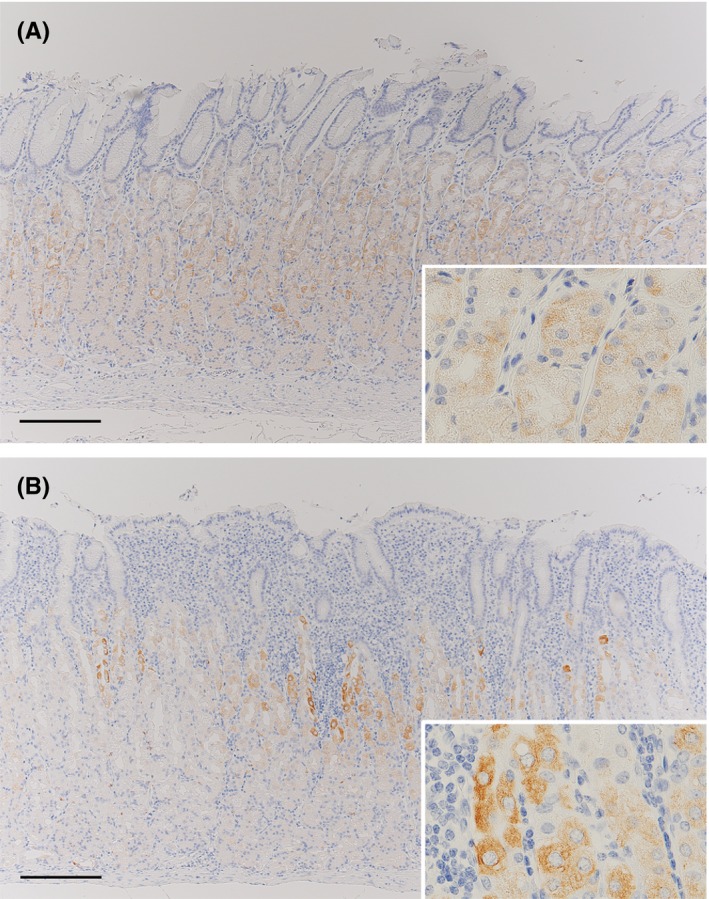
Representative images of CHAC1 IHC in gastric mucosa with or without *Helicobacter pylori* infection. (A) Weak positive signals of CHAC1 IHC (brown staining, see inset) are observed in the fundic‐gland mucosa without *H. pylori* infection. (B) Strong positive signals (see inset) and other weak positive signals (see inset) of CHAC1 IHC are observed in the fundic‐gland mucosa with *H. pylori* infection. Bars: 200 µm

**Figure 2:**
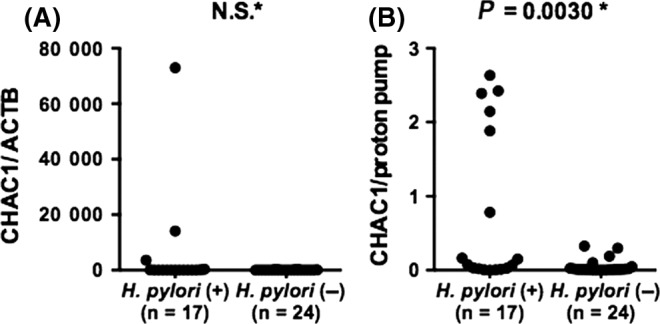
CHAC1 mRNA expression in gastric mucosa with or without *Helicobacter pylori* infection. (A) CHAC1 mRNA expression in fresh gastric mucosa samples normalized with that of β‐actin. (B) CHAC1 mRNA expression in the same samples normalized with that of the proton pump. *Mann‐Whitney *U* test. NS: not significant

Immunohistochemistry with the CHAC1‐mAb_(v1v2)_ revealed weak CHAC1 expression, ranging from a few to many cells, in the fundic‐gland areas of gastric mucosa with or without *H. pylori* infection (Figure 1A). Strong CHAC1 expression in a varying number of cells was observed in the fundic‐glands of gastric mucosa with *H. pylori* infection (Figure 1B). Fundic‐gland cells with transient density between the strong and weak signals developed by IHC were not infrequently observed in many samples with *H. pylori* infection and a few samples without the infection. CHAC1 mRNA expression was detected in all fresh gastric tissue samples, except for two samples without *H. pylori* infection. When CHAC1 mRNA expression in the fresh stomach samples was normalized to that of the housekeeping reference β‐actin gene, we observed no significant differences in relative CHAC1 mRNA expression between samples with and without *H. pylori* infection (Figure 2A). Considering the specific localization of strong CHAC1 expression in the parietal cells observed by IHC shown in Figure 1, we normalized CHAC1 mRNA expression to the human proton pump/H,K‐ATPase α subunit (proton pump) mRNA expression, which is specifically expressed in parietal cells. This analysis revealed a significant difference in CHAC1 mRNA expression between samples with and without *H. pylori* infection: Significant CHAC1 overexpression (ratio ranging from 0.8 to 2.6) was detected in 6 of 17 samples (35%) with *H. pylori* infection and in 0 of 24 samples without *H. pylori* infection (Figure 2B). Excluding the six infected samples with significant CHAC1 overexpression and the two uninfected samples with no CHAC1 expression detected, a constitutively low level of CHAC1 expression (ratio ranging from 0.001 to 0.1) was observed in all samples regardless of the *H. pylori* infection status, reflecting the weak CHAC1 expression detected by IHC in the fundic‐gland areas of gastric mucosa with and without *H. pylori* infection (Figure 1).

### 3.2 Localization of CHAC1 overexpression in *Helicobacter pylori*‐infected gastric mucosa

**Figure 3:**
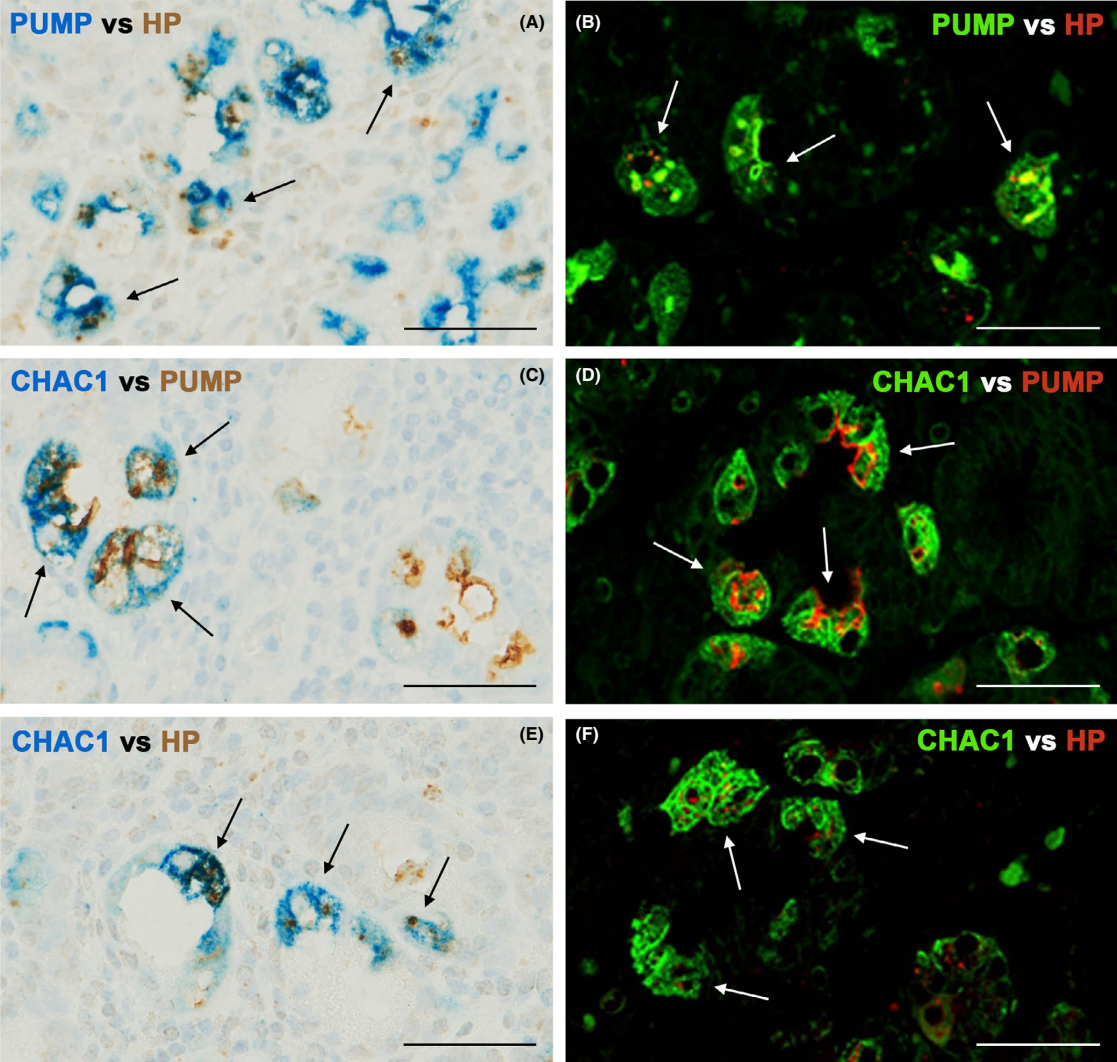
CHAC1 overexpression in proton pump/H,K‐ATPase α subunit (PUMP)‐positive parietal cells with *Helicobacter.pylori* (HP) infection. Double‐enzyme or fluorescence IHC for an identical sample of *H. pylori*‐infected fundic‐gland mucosa from a subject with chronic atrophic gastritis. *H. pylori* is located in the proton pump‐positive cells (arrows), (A and B) with anti‐proton pump antibody blue or green and TMDU‐mAb (anti‐*H. pylori*) brown or red. CHAC1 is overexpressed in the proton pump‐positive cells (arrows) (C and D) with CHAC1‐mAb blue or green and anti‐proton pump antibody brown or red. CHAC1 is overexpressed in parietal cells with *H. pylori* infection (arrows) (E and F) with CHAC1‐mAb blue or green and TMDU‐mAb brown or red. Bars: 50 µm

As CHAC1 seemed to be overexpressed in specific cells of the gastric mucosa with *H. pylori* infection, double‐enzyme or fluorescence IHC with each combination of two of the anti‐*H. pylori*, anti‐proton pump, and anti‐CHAC1 antibodies was performed using serial sections of the *H. pylori*‐infected stomach samples. *H. pylori* was detected in some of the parietal cells positive for expression of the proton pump (Figure 3A and 3). Strong CHAC1 expression was detected in some of these proton pump‐positive parietal cells (Figure 3C and 3). Strong CHAC1 overexpression was also observed in many *H. pylori*‐infected parietal cells (Figure 3E and 3). Taken together, these observations indicated that CHAC1 overexpression was localized in *H. pylori*‐infected parietal cells of the human gastric mucosa.

### 3.3 Immuno‐electron microscopy observation of *Helicobacter pylori* in parietal cells

**Figure 4:**
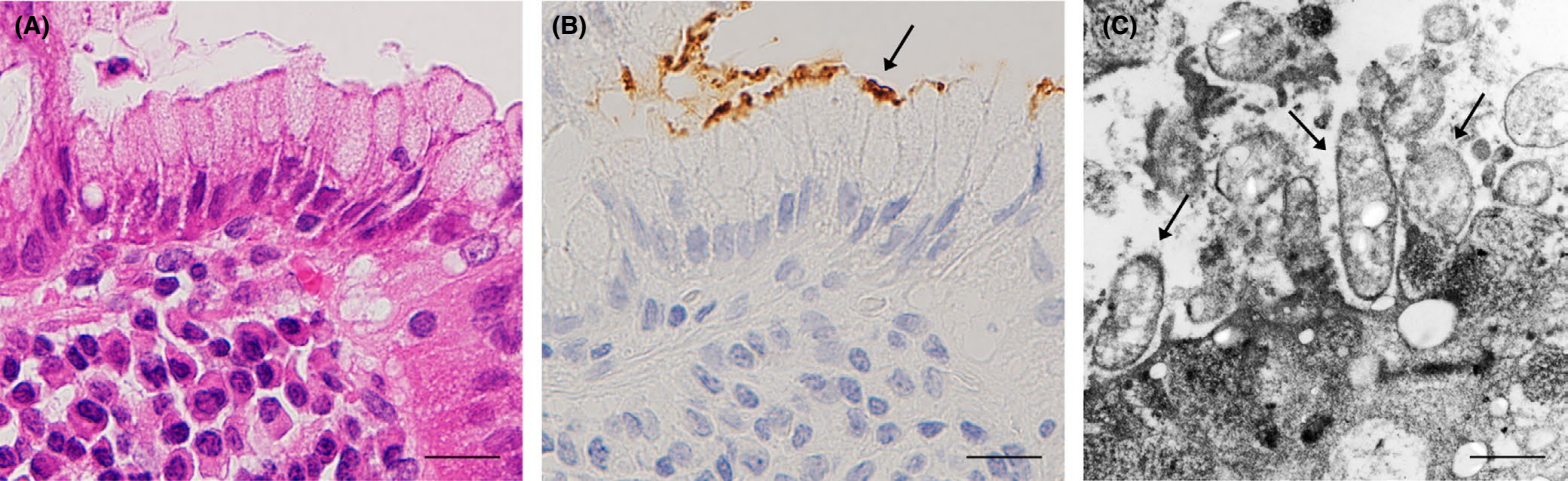
Localization of *Helicobacter pylori* cells in mucous layer attached to gastric foveolar epithelial cells. Serial histologic sections of a sample with prominent *H. pylori* infection were used for hematoxylin & eosin staining (A) and IHC with TMDU‐mAb (B) followed by immuno‐electron microscopy (C). (A and B) show an identical area of the *H. pylori*‐infected gastric mucosa. The area with immunoreactive *H. pylori* indicated by an arrow in b was subjected to immuno‐electron microscopy. (c) Several *H. pylori* organisms with a dense rim‐staining pattern indicated by arrows were observed on the foveolar epithelial cell. Bars: 20 µm (A and B), 1.0 µm (C)

**Figure 5:**
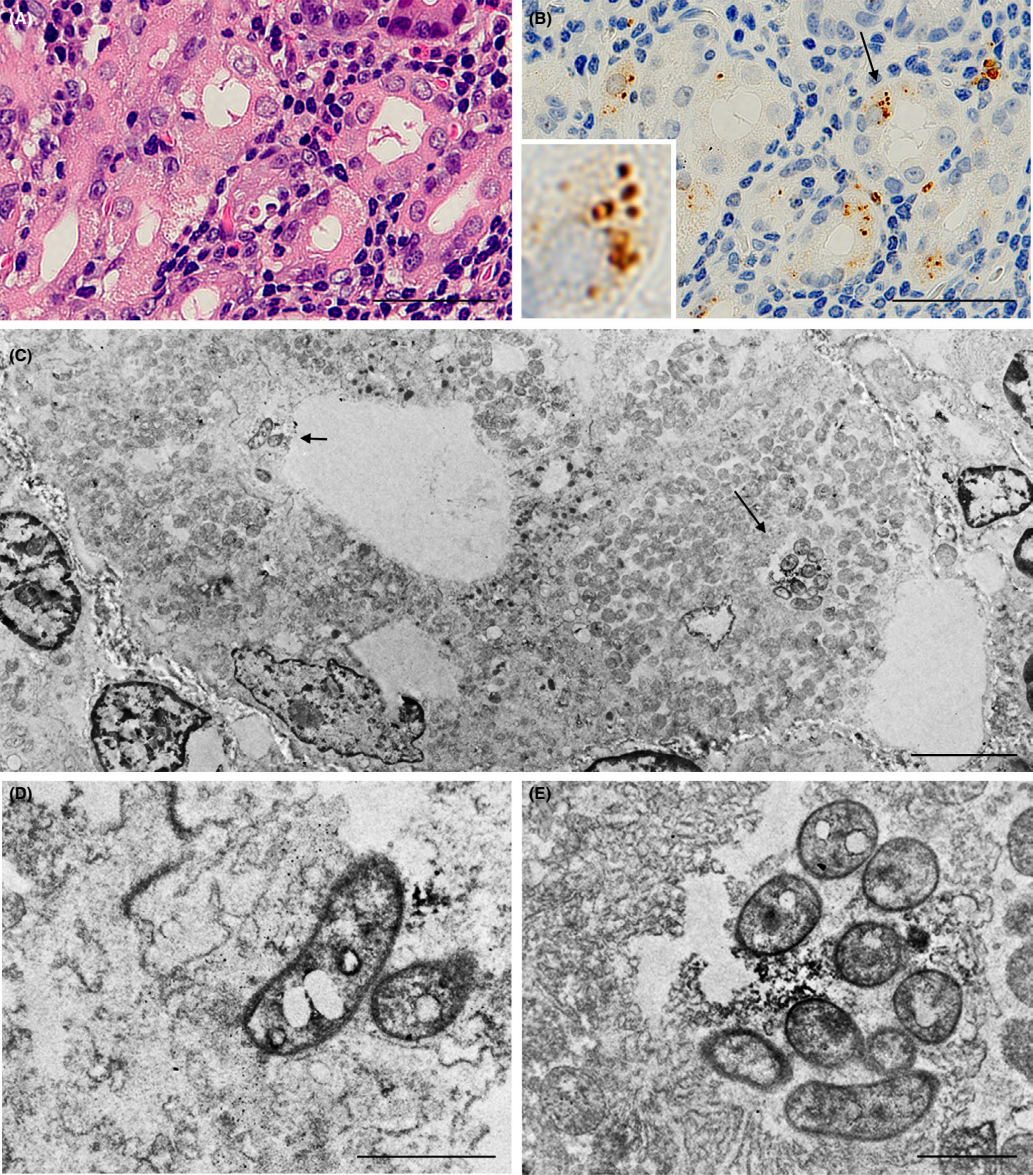
Localization of *Helicobacter pylori* cells in secretory canaliculi of the infected parietal cells. Serial histologic sections of a sample with prominent *H. pylori* infection in parietal cells were used for (A) hematoxylin & eosin staining; (B) IHC with TMDU‐mAb (anti‐*H. pylori*); followed by (C) immuno‐electron microscopy. In b an arrow indicates *H. pylori* colonization and an inset shows higher magnification of the bacteria indicated by the arrow. (C) *H. pylori* colonization was observed in the secretory canaliculi of the parietal cells and is located near the entry (a short arrow) and in a deeper area (long arrow). (D and E) respectively show higher magnification of the *H. pylori* cells indicated in c by a short or long arrow. Note the intact and not denatured bacterial cells with a dense rim‐staining pattern corresponding to the distribution of the bacterial cell membrane‐bound lipopolysaccharide detected by the antibody. Bars: 50 µm (A and B), 5.0 µm (C) and 1.0 µm (D and E)

Although *H. pylori* is generally considered a noninvasive extracellular bacterium, many immunoreactive signals were detected inside some parietal cells in many samples with *H. pylori* infection. Therefore, we used immuno‐electron microscopy to further examine the localization of *H. pylori* in the gastric mucosa (Figure 4A and B, and Figure 5A and 5). *Helicobacter pylori* staining in the mucous layer revealed dense rim staining of a whole *H. pylori* organism, consistent with the assumed distribution of lipopolysaccharides (Figure 4C). In addition, some bacterial cells that had the same staining patterns were located in the secretory canaliculi of the parietal cells (Figure 5C). Higher magnification confirmed that the *H. pylori* cells embedded in the secretory canaliculi were intact (Figure 5D and 5).

### 3.4 Evidence of proliferation found in some parietal lineage cells

**Figure 6:**
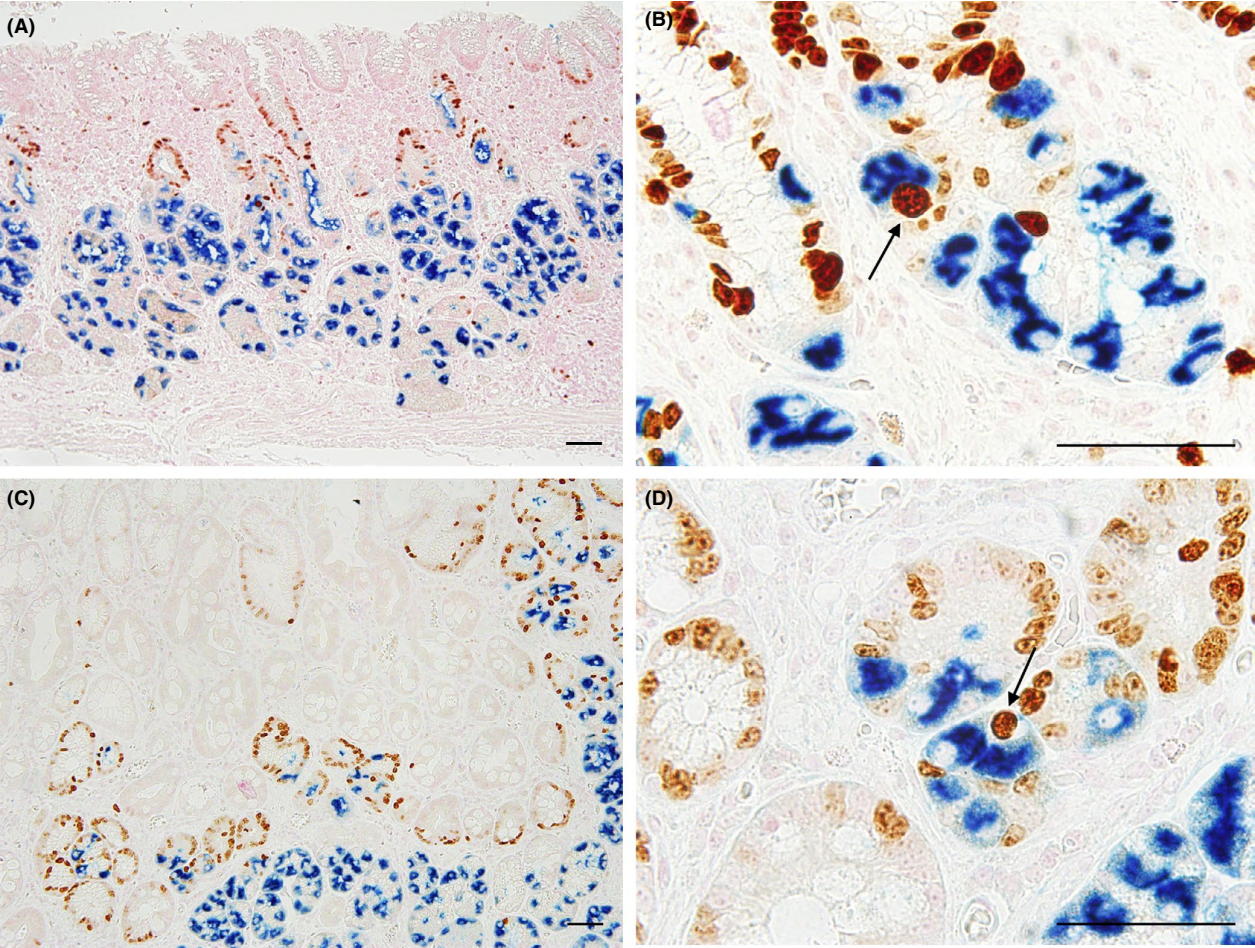
Proliferating parietal cells in gastric mucosa with *Helicobacter pylori* infection. Double‐enzyme IHC with anti‐proton pump antibody (blue) and anti‐Ki67 antibody (brown) was performed on samples with non‐atrophic superficial gastritis (A and B) or chronic atrophic gastritis (C and D): Non‐atrophic superficial gastritis samples exhibited many cells with nuclear signals of Ki67 in the neck zone (A and B). In chronic atrophic gastritis samples, many proton pump‐ and Ki 67‐positive cells were identified in a deeper area (C and D). Proton pump‐positive parietal cells showing nuclear Ki67 staining are indicated by arrows in (B and D). Bars: 50 µm

*Helicobacter pylori* infection contributes to the progression of gastric cancer.^16^, ^17^, ^18^ *Helicobacter pylori* infection induces changes to the gastric mucosa and increases cell proliferation, which can eventually lead to inflammation‐associated oncogenesis.^19^ We examined the extent of parietal cell proliferation in *H. pylori*‐infected samples using double‐enzyme IHC with anti‐Ki67 antibody to detect proliferation and an anti‐proton pump antibody to identify parietal cells. These studies indicated that some parietal cells exhibited positive nuclear signals for Ki67 in the neck zone of the gastric fundic‐gland mucosa with *H. pylori* infection (Figure 6).

## 4 DISCUSSION

Cation transport regulator 1 is a novel ER stress‐inducible gene, and in the presence of ER stress, CHAC1 mRNA levels are upregulated.^3^, ^4^ Infection is a factor that stimulates ER stress,^20^, ^21^, ^22^ and our previous in vitro study demonstrated that *H. pylori*‐triggered ER stress led to the overproduction of CHAC1.^12^ To date, there have been no IHC studies of CHAC1 expression in human tissues because commercially available anti‐CHAC1 antibodies cannot be used in FFPE samples. Using a novel antibody against CHAC1,^12^ however, we demonstrated in situ CHAC1 expression in human stomach FFPE samples. CHAC1 expression was observed as weakly stained positive signals in fundic‐gland areas of the gastric mucosa irrespective of the *H. pylori* infection status. In contrast, *H. pylori*‐infected samples showed strongly stained CHAC1‐positive signals in some fundic‐gland cells. Colocalization of proton pump signals with CHAC1 expression suggests that *H. pylori* infection is specifically associated with CHAC1 overexpression in the parietal cells in the stomach. These findings confirmed in vivo the previously reported association between *H. pylori* infection and the strong induction of CHAC1. This confirmation suggests that, in contrast to the low‐level constitutive expression of CHAC1 observed in many fundic‐gland cells, high levels of CHAC1 overexpression observed in some parietal cells are likely to induce the remarkable changes in the cellular redox balance and increase in *TP53* mutations^12^ that are associated with the CHAC1 overexpression induced by *H. pylori* infection in vitro.

The TMDU‐mAb used in this study has higher sensitivity for detecting *H. pylori* in FFPE samples of the human stomach than commercially available anti‐*H. pylori* antibody products, such as the polyclonal antibody from DAKO (B0471) and the monoclonal antibody from CHEMICON (MAB922), as described previously.^14^ IHC with TMDU‐mAb can detect *H. pylori* not only in the mucous layer, but also in macrophages and parietal cells of *H. pylori*‐infected gastric mucosa. Notably, double‐enzyme IHC revealed no CHAC1 overexpression in gastric foveolar epithelial cells, even in cells with many *H. pylori* attached to the surface. In contrast, CHAC1 overexpression was observed by IHC in parietal cells of the *H. pylori*‐infected stomach, including all cases with significant CHAC1 mRNA overexpression. To investigate the localization of *H. pylori* in parietal cells, we performed immuno‐electron microscopy. In *H. pylori*‐infected gastric mucosa, bacterial cells were not observed in the cytoplasmic space, but in the secretory canaliculi of the parietal cells. These bacteria were morphologically similar to those observed in the mucous layer and, based on their rim staining, the immunoreactive bacterial cells appear to remain intact. This is consistent with the lipopolysaccharide distribution of whole *H. pylori* organisms in the mucous layer.^14^ *Helicobacter pylori* infection affects epithelial cells at an early stage when a large number of *H. pylori* cells are present,^16^ whereas the direct actions of *H. pylori* on epithelial cells seem to be less important at the late stage when the *H. pylori* cells appear to decrease in number or even disappear altogether with the progression of atrophic gastritis in association with the development of intestinal metaplasia.^16^ The present findings suggest that stable *H. pylori* colonization in the secretory canaliculi of parietal cells, rather than the presence of unstably attached *H. pylori* to foveolar epithelial cells, may be necessary to induce CHAC1 overexpression. The reason why *H. pylori* can live in the secretory canaliculus of the parietal cell, where the pH is extremely low,^23^ may be associated with *H. pylori*‐mediated repression of the proton pump, the parietal cell enzyme mediating acid secretion, and ensuing hypochlorhydria in the secretory canaliculus of the infected parietal cell.^24^

Previous in vitro experiments identified CHAC1 overexpression as a risk factor for the induction of nucleotide alterations in the *TP53* tumor suppressor gene resulting from cellular oxidative stress, which initially arises from the depletion of intracellular GSH by CHAC1 and the accumulation of ROS.^9^, ^10^, ^11^, ^12^, ^25^, ^26^ Thus, stable *H. pylori* colonization in the secretory canaliculi of parietal cells may lead to constant CHAC1 overexpression in those cells, resulting in genetic mutations that are more likely to contribute to gastric carcinogenesis. Because it has not been feasible to evaluate CHAC1 activity in histologic samples, further understanding of the CHAC1 function in specific cell types will depend on future technical developments.

*Helicobacter pylori* infection is a leading factor in the development of gastric carcinoma.^16^, ^17^, ^18^ During *H. pylori* infection, several alterations occur in the gastric mucosa, including cell proliferation, and these changes eventually lead to inflammation‐associated oncogenesis.^19^ *TP53* mutations induced by CHAC1 overexpression may be more likely to contribute to gastric carcinogenesis in the parietal cells of the gastric neck zone with a long cell cycle (54 days) than in the foveolar epithelial cells of the gastric‐pit zone with a short cell cycle (3 days).^27^ Stem cells of the gastric mucosa give rise to the committed progenitors of parietal cells (pre‐parietal cells), either directly or indirectly.^28^ Indeed, in the present study, some proton pump‐positive cells of parietal cell lineage were in the process of cell proliferation as indicated by positive Ki67 staining. Thus, *H. pylori*‐infected parietal cells with the potential for survival and proliferation may be key contributors to the development of gastric cancer.

Gastric adenocarcinoma is a heterogeneous disease commonly classified into two main histologic types, intestinal and diffuse.^29^ *Helicobacter* infection is a major risk factor for both types.^30^ *Helicobacter pylori* infection is associated with the development of diffuse‐type gastric cancer,^31^, ^32^ which is thought to develop directly from chronic gastritis without intestinal metaplasia, whereas intestinal‐type gastric cancer is thought to develop from atrophic gastritis with intestinal metaplasia.^33^, ^34^, ^35^ Gastric cancer arising specifically from parietal cells has not been extensively investigated. Shimada et al^15^ reported diffuse‐type gastric cancer in all double‐conditional knockout mice in which E‐cadherin and p53 were specifically inactivated in the gastric parietal cell lineage. Thus, DNA damage to these genes by CHAC1‐induced oxidative stress in the gastric parietal cell lineage is a potential mechanism leading to the development of diffuse‐type gastric cancer in patients with *H. pylori* infection.

In conclusion, CHAC1 overexpression was induced in parietal cells of the gastric mucosa where *H. pylori* stably colonized in their secretory canaliculi. Taken together with the results from our previous study in vitro,^12^ *H. pylori*‐induced CHAC1 overexpression in parietal cells may cause somatic mutations that further contribute to the development of gastric cancer.

## DISCLOSURE OF INTERESTS

No competing interests declared.

## AUTHOR CONTRIBUTIONS

TO and YW designed the study design, performed most of the experiments, analyzed and interpreted the data, and wrote the manuscript. PGB contributed to the study design and helped write the manuscript. KT and TS contributed to the study design. KU, KK, TT, YT, and YN provided the study material and technical support. YE supervised and directed the project, and contributed to the manuscript preparation.
