# Supplementary material for: CHAC1 overexpression in human gastric parietal cells with Helicobacter pylori infection in the secretory canaliculi
Source: Helicobacter. 2019 May 20;24(4):e12598. doi: 10.1111/hel.12598 (PMC6618068; doi:10.1111/hel.12598)
Supplement: Supplementary file 1 [file HEL-24-na-s001.pdf]

Supplementary Information

CHAC1 overexpression in human gastric parietal cells with *Helicobacter pylori* infection in the secretory canaliculi

Tomohisa Ogawa[¶], Yuriko Wada[¶], Kosuke Takemura, Philip G Board,

Keisuke Uchida, Keisuke Kitagaki, Tomoki Tamura, Takashige Suzuki,

Yutaka Tokairin, Yasuaki Nakajima & Yoshinobu Eishi[*]

Supplementary Table 1 Clinical profiles of patients with esophageal cancer from whom fresh and formalin-fixed tissues of the gastric mucosa were obtained.

Patient no.	Age	Gender	Histologic diagnosis	*H. pylori*		
				IHC	real-time PCR	serum IgG [U/ml]
H. pylori-positive patients						
1	66	M	Squamous cell carcinoma of the esophagus	+	39211	29
2	73	M	Squamous cell carcinoma of the esophagus	+	28000	34
3	67	M	Adenocarcinoma in the Barrett esophagus	+	23510	14
4	66	F	Squamous cell carcinoma of the esophagus	+	12716	5
5	69	M	Squamous cell carcinoma of the esophagus	+	6913	37
6	63	M	Squamous cell carcinoma of the esophagus	+	5569	83
7	68	M	Squamous cell carcinoma of the esophagus	+	5040	N.E.
8	59	F	Squamous cell carcinoma of the esophagus	+	3506	8
9	73	M	Squamous cell carcinoma of the esophagus	+	1237	17
10	74	M	Squamous cell carcinoma of the esophagus	+	170	41
11	64	M	Squamous cell carcinoma of the esophagus	+	117	15
12	70	M	Squamous cell carcinoma of the esophagus	+	110	< 3
13	73	M	Adenocarcinoma at the esophagogastric junction	+	107	N.E.
14	67	M	Squamous cell carcinoma of the esophagus	+	85	49
15	62	M	Squamous cell carcinoma of the esophagus	+	30	20
16	69	M	Adenocarcinoma at the esophagogastric junction	+	27	< 3
17	65	F	No carcinoma, remaining in the operative material	+	7	< 3
H. pylori-negative patients						
18	76	M	Squamous cell carcinoma of the esophagus	-	0	7
19	57	M	Squamous cell carcinoma of the esophagus	-	0	4
20	61	M	Squamous cell carcinoma of the esophagus	-	0	4
21	51	M	Squamous cell carcinoma of the esophagus	-	0	< 3
22	58	M	Adenocarcinoma at the esophagogastric junction	-	0	< 3
23	74	M	Squamous cell carcinoma of the esophagus	-	0	< 3
24	71	M	Adenocarcinoma at the esophagogastric junction	-	0	< 3
25	62	M	Squamous cell carcinoma of the esophagus	-	0	< 3
26	60	M	Squamous cell carcinoma of the esophagus	-	0	< 3
27	71	F	Squamous cell carcinoma of the esophagus	-	0	< 3
28	73	M	Squamous cell carcinoma of the esophagus	-	0	< 3
29	57	M	Multiple Squamous cell carcinoma of the esophagus	-	0	< 3
30	49	M	Squamous cell carcinoma of the esophagus	-	0	< 3
31	61	M	Multiple Squamous cell carcinoma of the esophagus	-	0	N.E.
32	68	M	Adenocarcinoma at the esophagogastric junction	-	0	N.E.
33	78	M	Squamous cell carcinoma of the esophagus	-	0	N.E.
34	52	M	Squamous cell carcinoma of the esophagus	-	0	N.E.
35	74	M	Squamous cell carcinoma of the esophagus	-	0	N.E.
36	55	M	No malignancy in the examined specimen of esophagus	-	0	N.E.
37	70	M	Adenocarcinoma at the esophagogastric junction	-	0	N.E.
38	57	F	Squamous cell carcinoma of the esophagus	-	0	N.E.
39	54	M	Adenocarcinoma at the esophagogastric junction	-	0	N.E.
40	62	M	Squamous cell carcinoma of the esophagus	-	0	N.E.
41	53	M	Squamous cell carcinoma of the esophagus	-	0	N.E.

The enzyme IHC results for *H. pylori* were considered positive when any positive signals were identified in the mucous layers or lamina propria of the gastric mucosa. The real-time PCR number is shown as the total number of *H. pylori* in the sample. N.E.: not examined.

Supplementary Table 2 Primers and probes used in this study

Primers	Orientation	Sequence (5’ – 3’)
human *CHAC1*	Sense	GGTGACGCTCCTTGAAGATCAT
	Antisense	TCAGTGGTTGGTCAGGAGCAT
	Probe	[FAM]-AGAGGCAGTGCTTGGTGGCTACGATACC-[TAMRA]
16S rRNA of *H. pylori*	Sense	GGGCTTA GTCTCTCCAGTAATGCA
	Antisense	GAGTTTTA ATCTTGCGACCGTACTC
	Probe	[FAM]-CTAACGCATTAAGCATCCCGCCTGG-[TAMRA]
human *β-actin*	Sense	GGATGCAGAAGGAGATCACTG
	Antisense	CGATCCACACGGAGTACTTG
	Probe	[TET]-CCCTGGCACCCAGCACAATG-[TAMRA]
human *proton pump/H,K-ATPase α subunit*	Sense	CAACTTTGCCTCCAT
	Antisense	TGGAGCAGGGTCGACTGATCTTCGA
	Probe	[FAM]-TGAAGAAGTCTATTGCCTACACATTGA-[TAMRA]
